# A functional polymorphism in the DNA methyltransferase-3A promoter modifies the susceptibility in gastric cancer but not in esophageal carcinoma

**DOI:** 10.1186/1741-7015-8-12

**Published:** 2010-02-03

**Authors:** Hong Fan, Dongsheng Liu, Xuemei Qiu, Fengchang Qiao, Qingxiang Wu, Xianwei Su, Feng Zhang, Yunwei Song, Zhujiang Zhao, Wei Xie

**Affiliations:** 1 Key Laboratory of Developmental Genes and Human Diseases, Ministry of Education, Southeast University, Nanjing 210009, China; 2 Institute of Life Science, Southeast University, Nanjing 210009, China; 3 Clinical laboratory, Suqian People's Hospital, Suqian, 223800, China

## Abstract

**Background:**

DNA-methyltransferase (DNMT)-3A plays an important role in the development of embryogenesis and the generation of aberrant methylation in carcinogenesis. The aim of this study was to investigate the role of a DNMT3A promoter genetic variant on its transcriptional activity and to evaluate the association between DNMT3A gene polymorphism and the susceptibility to gastric cancer (GC) and oesophagus carcinoma (EC) in the Chinese population.

**Methods:**

We selected one of the single nucleotide polymorphisms (SNPs) -448A>G in the DNMT3A promoter region and evaluated its effect on activity using a luciferase assay. -448A>G polymorphisms of DNMT3A were determined by polymerase chain reaction/restriction fragment length polymorphism and confirmed by sequencing. The distribution of -448A>G polymorphisms was detected in 208 GC patients and 346 healthy controls matched for age and gender. The distribution of -448A>G polymorphisms was also detected in 96 EC patients and matched 241 healthy controls. The association of -448A>G polymorphisms of DNMT3A and the risk of GC and EC was evaluated by stratified analysis according to the patient's age and gender.

**Results:**

In a promoter assay, carriage of the -448 A allele showed a significantly higher promoter activity (> two fold) compared with the -448G allele (*P *< 0.001). The allele frequency of -448A among GC patients and controls was 32.9% versus 19.9%, respectively. Overall, we found that, compared with GG carriers, the DNMT3A -448AA homozygotes has a > six fold increased risk of GC. Stratification analysis showed that AA homozygotes have a more profound risk in the subgroups of individuals at the age range ≤ 60 years in GC. However, individuals with -448AG and -448AA were not statistically significantly associated with an increased risk of EC compared with those carried the -448GG genotype.

**Conclusions:**

The DNMT3A -448A>G polymorphism is a novel functional SNP and contributes to its genetic susceptibility to GC. -448A>G can be used as a stratification marker to predict an individual's susceptibility to GC, especially in the subgroups of individuals at the age range ≤ 60 years. However, the relative distribution of -448A>G in EC can not be used as a prediction marker in order to evaluate an individual's susceptibility to EC.

## Background

Abnormal DNA methylation is thought to be a major early event in the development of tumours where DNA methyltransferases (DNMTs), DNMT1, DNMT3A and DNMT3B have been identified as DNA methylation functional enzymes in eukaryotic cells [1,2]. DNMT1, which is often referred to as the maintenance methyltransferase, is responsible for maintaining pre-existing methylation patterns during DNA replication [3,4]. DNMT3A and DNMT3B are considered to be *de novo *DNA methyltranferase, which are critical in the dynamic DNA methylation process during embryogenesis and pathogenesis [5-7]. Aberrant promoter methylation in various tumour suppressor genes is also involved in human gastric cancer and oesophagus carcinoma [8-10] and the epigenetic silencing linked this aberrant *de novo *methylation of CpG islands to the overexpression of the DNMT-3 family (DNMT3A and DNMT3B). Studies showed that DNMT3A and DNMT3B, like DNMT1, repress transcription in a methylation-dependent manner [11,12]. Recently, Bachman *et al*. [13] showed that DNMT3A and DNMT3B repress the transcription independence of their methylating activities and that this repression is partially dependent upon histone deacetylase activity. DNMT3A associates with the histone deacetylase HDAC1 using its ATRX-homology domain as a co-repressor for RP58 [14].

Numerous studies have shown DNMT overexpression in a variety of cancers and that it maybe involved in carcinogenesis [15,16]. Studies on the depletion of DNMT1 and DNMT3B in tumour cells have implied that they play an important role in tumour development. DNMT3A is essential for mammalian development and is responsible for the generation of genomic methylation patterns. Samuel *et al*. used knock-in transgenic mice in order to investigate the consequences of intestinal epithelium-specific overexpression of *de novo *DNMT3A [10]. Until then it was unclear what part DNMT3A expression and its biological function played in tumors. The study showed that an elevated DNMT3A expression was consistent with a repressed imprinting gene SFRP5 and promoted polyposis in *APC *Min mice. DNMT3A expression in gastric cancer (GC) has only been studied in protein levels and showed a significant overexpression of DNMT3A in tumours while DNMT1 and DNMT3B were only modestly over-expressed [17]. DNMT3A expression and its clinical significance in oesophagus carcinogenesis is uncertain.

Many gene polymorphisms, including some epigenetic marker genes, have been reported to be closely associated with a susceptibility to tumours. The polymorphism in the DNMT3B promoter that plays a role in *de novo *methylation has also been reported to be associated with several tumour susceptibilities, including gastric cancer [18,19]. However, few studies have reported on the possible association of cancer susceptibility with DNMT3A, another active *de novo *methyltransferase in mammals. To our knowledge, the association between DNMT3A polymorphisms and clinical implication of GC and esophageal carcinoma (EC) has not been previously been reported. In the present work, we want to determine whether a single nucleotide polymorphism (SNP) in DNMT3A promoters contributes to its increased expression and whether functional SNP is substantially associated with cancer susceptibility. We now show that, as with some SNPs in the DNMT3B promoter, a SNP in DNMT3A promoter could activate its expression at the transcription level. However, it is important to know whether the transcriptional activation of the SNP of DNMT3A is related to tumourigenesis. We hypothesized that the genetic variants of DNMT3A that are responsible for regulating the methylation status of other genes are associated with an increased risk of cancer. In this hospital-based case-control study, we genotyped a functional DNMT3A polymorphism and investigated the association between this genetic variant and the risk of gastric cancer and oesophagus carcinoma.

## Methods

### Study subjects

We recruited 208 GC patients, 96 EC patients and 346 healthy individuals (controls). Tissue samples for immunohistochemical analysis were taken from 56 GC patients. All patients were confirmed histopathologically and samples were obtained with informed consent from the patients and the controls and the approval of the institutional review board at the Zhongda Hospital of Southeast University and Jiangsu Tumor Hospital in Jiangsu province from September 2006 to June 2008. The controls were selected from cancer-free individuals who visited the same hospital for regular physical examinations or who volunteered to participate in the epidemiology survey during the same period. We defined a healthy volunteer as a someone who was seen to free from disease (including a cancer-free history) at a health check-up. The controls were matched for age and gender with the patients (Tables 1 and 2). All patients and controls were ethnically Chinese and resided in the Jiangsu Province of China or its surrounding regions.

**Table 1:** Characteristics of the gastric cancer (GC) study population

Variables	Controls (*n *= 346)	GC cases (*n *= 208)	*P *value †
Age (years)			0.115
≤60	141 (40.8)*	99 (47.6)	
>60	205 (59.2)	109 (52.4)	
Gender			0.182
Male	243 (70.2)	157 (75.5)	
Female	103 (29.8)	51 (24.5)	

*Numbers in parentheses, percentage.

† *P *value for chi-squared test.

**Table 2:** Characteristics of the esophageal cancer (EC) study population

Variables	Controls (*n *= 241)	EC cases (*n *= 96)	*P *value†
Age (years)			0.395
≤60	141 (58.5)*	61 (63.5)	
>60	100 (41.5)	35 (36.5)	
Gender			0.177
Male	160 (66.4)	71 (74.0)	
Female	81 (33.6)	25 (26.0)	

*Numbers in parentheses, percentage.

† *P *value for chi-squared test.

### SNP selection and luciferase assay

Among the candidate SNPs in DNMT3A, we focused on an SNP in the promoter region (rs 1550117 in promoter; GenBank accession No. NT_022184.14:g.4381840 A/G). The fragments of the DNMT3A promoter region (from -1009 to +646, transcription start site of exon 1A counted as t1) was synthesized by polymerase chain reaction (PCR) using genomic DNA from donors carrying either the wild-type or polymorphic allele of DNMT3A promoter region. The PCR primers used for the exon 1A promoter regions was 5'-GGAGGGACCTGGAAGCATTG-3' (forward) and 5'-TTACCGTATGGCCGGTGGG-3' (reverse). The PCR products were inserted upstream of the luciferase gene in the pGL3-basic plasmid (Promega, Madison, WI), and the correct sequence of all clones was verified by DNA sequencing. Promoter activity was measured using the Lucifierase Reporter Assay System (Promega, WI, USA). Chinese hamster ovary (CHO) cells were grown in minimal essential medium supplemented with 10% fetal bovine serum. 5 × 10^5 ^cells were plated in a six-well plate and transfected with DNMT3A promoter SNP fragments and empty vector pGL3-basic using Lipofectamine 2000™ (Invitrogen, CA, USA). Cells were harvested 48 h post-transfection followed by the luciferase reporter assay in the cell lysate. The experiment was performed five times in triplicates and the results were reported as mean ± standard deviation (SD).

### DNMT3A genotyping

Samples were collected into blood vacuum tubes containing ethylenediaminetetra-acetic acid (EDTA) and stored at 4°C. Genomic DNA was extracted within 1 week of sample collection by proteinase K digestion as previously described [20]. The transition of A>G of DNMT3A SNP creates a *Taa*I restriction site, PCR-restriction fragment length polymorphism (RFLP) was used to detect this A-G transition in the promoter of DNMT3A at -448A>G (GenBank accession No. NT_022184.14:g.4381840). The DNMT3A -448A>G polymorphisms was determined by a PCR-RFLP assay. The PCR reaction was performed in a total of 25ìL containing 100 ng genomic DNA, 0.1 mM dNTPs, 2.0 mM MgCl2, 10ìM primers 5'-ACACACCGCCCTCACCCCTT-3' (forward), and 5'-TCCAGCAATCCCTGCCCACA-3' (reverse), and 1.25 U Taq polymerase (Biocolor BioScience and Technology Co, Shanghai, China). PCR cycle conditions consisted of an initial melting step of 95°C for 5 min, followed by 32 cycles of 95°C for 20s, 66°C for 20s, 72°C for 20s and a final extension step of 72°C for 10 min. The 358 bp fragment was then digested with *Taa*I (Takara Biotechnology Co. Ltd, Dalian, China) 2 h at 65°C, the digested products were separated on a 2.0% agarose gel and the RFLP bands visualized under ultraviolet light with ethidium bromide (EB) staining. The wild-type G allele consists of a *Taa*I restriction site that results in three bands (153 bp, 94 bp and 87 bp), while the variant A allele produces four bands (247 bp, 153 bp, 94 bp and 87 bp). For quality control, genotyping analysis was performed blind, with respect to case/control status, and repeated twice for all subjects. The results of genotyping were 100% concordant. In order to confirm the genotyping results, randomly selected PCR-amplified DNA samples (*n *≥ 3, respectively, for each genotype) were examined by DNA sequencing, and the results were also 100% concordant.

### Immunohistochemistry

We deparaffinized and dehydrated 4 μm-thick sections of formalin-fixed, paraffin embedded tissue specimens from all 56 patients. For antigen retrieval, the sections were heated for 10 min at 120°C in an autoclave and nonspecific reactions were blocked with 5% normal horse serum. All sections were incubated with specific primary antibodies that recognized DNMT3A mouse monoclonal antibody dilution 1:75 (Imgenex, IMG-268A) and followed by incubation with biotinylated secondary antibodies (anti-goat IgG, anti-mouse IgG, dilution 1:200; Qiagen Laboratories, CA, USA) at room temperature for 30 min. The sections were then treated with Vectastain Elite ABC reagent (Vector Laboratories, CA, USA). All sections were counterstained with haematoxylin. For negative control preparations, the primary antibody was omitted from the reaction sequence.

### Statistical analysis

Patients and controls were compared using Student's *t*-test for continuous variables and chi square (χ^2^) test for categorical variables. The Hardy-Weinberg equilibrium was tested with a goodness-of-fit χ^2 ^test and 1° freedom to compare the observed genotype frequencies and the expected genotype frequencies among subjects. A comparison of the DNMT3A genotype and allele distributions among the study groups was performed by means of two-sided contingency tables using χ^2 ^test or Fischer's exact test. The odds ratio (OR) and 95% confidence interval (CI) were calculated using the STATA 10.0 software (Stata Corp, Texas, USA). *P *< 0.05 was considered statistically significant.

## Results

### Identification of a novel functional DNMT3A polymorphism

**Figure 1:**
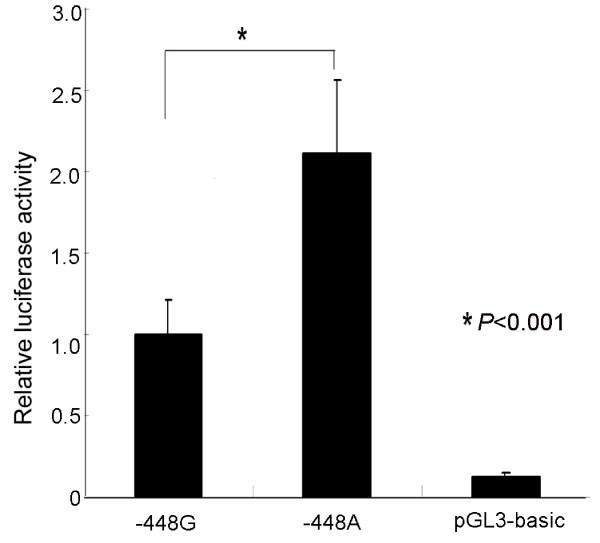
**Transcription activity analysis of the -448G>A polymorphism of DNMT3A promoter**. Chinese hamster ovary cells were transiently transfected with luciferase reporter constructs. The pGL3-basic plasmid lacking promoter sequence was used as a negative control. The data represents mean -- standard deviation calculated from four independent experiments performed in triplicates and are expressed as a percentage of the -448A allele activity. The difference between the -448G and -448A constructs was significant at *P *< 0.001 (*t*-test; *n *= 6).

Several candidate SNPs in the DNMT3A gene have been deposited in public databases http://www.ncbi.nlm.nih.gov/SNP. Although the functional effects of these polymorphisms have not been elucidated, we hypothesized that some of these variants may influence DNMT3A activity on DNA methylation, especially those located at the promoter region of the gene. We choose and investigated the effects of the -448A>G polymorphism on the promoter activity of DNMT3A by luciferase assay. The promoter activity of the -448A allele was significantly higher (more than double) compared with the -448G allele (*P *< 0.001, Figure 1).

### Genotyping of SNPs

**Figure 2:**
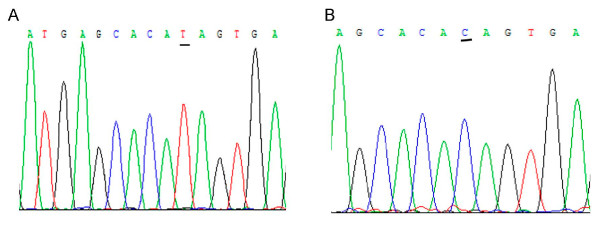
**Sequencing results for the polymerase chain reaction (PCR) products from different -448G>A genotypes**. The single nucleotide polymorphism sites are underlined. The results were completely matched to the corresponding results derived from PCR-restriction fragment length polymorphism genotyping. A: AA variants (reverse strand), B: GG wild type (reverse strand).

The -448A>G polymorphism in the promoter of DNMT3A gene was first investigated in the Chinese healthy control and patients with GC and EC by PCR-RFLP. The DNMT3A genotypes AA, AG, and GG were detected in the GC and EC patients and the controls. The genotyping by PCR-RFLP analysis was completely confirmed by DNA sequencing analysis (Figure 2). The distributions of -448A>G genotypes in 346 healthy controls are GG 63.0%, GA 34.1%, AA 2.9%, and A allele frequency is 19.9%, shown in Table 3. The -448A>G polymorphism of DNMT3A promoter was evaluated and the risk related to GC groups in the case-control study. There were no significant differences in the mean age and gender distribution between cases and controls, suggesting that the matching based on these two variables was adequate (Table 1). The mean age was 65 years (range, 34~80 years) for the GC patients and 71 years (range, 32~80 years) for the control subjects. All patients and controls were successfully genotyped for the DNMT3A polymorphism. The DNMT3A -448A>G polymorphism was distributed in the Hardy-Weinberg equilibrium.

**Table 3:** *DNMT3A *-448A>G genotype and allele frequencies in control subjects

Groups	Genotype			Allele	
	GG (%)	AG (%)	AA (%)	A (%)	P *
Total	218 (63.0)	118 (34.1)	10 (2.9)	19.9	
Age years)					0.09^†^
≤60	80 (23.1)	57 (16.5)	4 (1.2)	9.4	
>60	138 (39.9)	61 (17.6)	6 (1.7)	10.5	
Gender					0.521^‡^
Male	152 (43.9)	82 (23.7)	9 (2.6)	14.5	
Female	66 (19.1)	36 (10.4)	1 (0.3)	5.5	

*Chi-squared test.

† The frequency of A allele in individuals at ≤ 60 years versus > 60 years.

‡ The frequency of A allele in individuals in male versus female.

The gastric cancer risks related to the DNMT3A -448A>G genotype are shown in Tables 4 and 5. The allele frequency of -448A among GC patients and controls was 32.9% versus 19.9%, respectively. The distributions of -448A>G genotypes in the GC group (GG 49.0%, AG 36.1%, AA 14.9%) were significantly different from those among the controls (*P *= 0.000). The OR and their 95% CI were calculated using the more common homozygous variant genotype as the reference group (-448 GG genotypes). Compared to reference group, AA homozygotes had a >six fold increased risk of GC (OR 6.625; 95% CI = 3.128-14.034, *P *= 0.000). When the analyses were stratified by age and gender of patients, the AA genotype was associated with a significantly increased risk of GC (OR 9.500, 95% CI = 3.029-29.796 *P *= 0.000) at ≤ 60 years and more 2.13-fold at >60 years (OR 4.452, 95% CI = 1.598-12.404 *P *= 0.002). The AA genotype was associated with a significantly increased risk of GC (OR 10.313, 95% CI = 1.156-91.974 *P *= 0.012) in the females and was more 1.6-fold greater than in males (OR 6.273, 95% CI = 2.793-14.089 *P *= 0.000).

**Table 4:** DNMT3A -448A>G genotype and allele frequencies of case patients and control subjects and their association with gastric cancer (GC)

Genotype	GC (*n *= 208)		Control subjects (*n *= 346)		Crude odds ratio (95% confidence interval)	*P *value*
				
	**No**.	(%)	**No**.	(%)		
- 448A>G						
GG (ref.)	102	(49.0)	218	(63.0)	1.0	
AG	75	(36.1)	118	(34.1)	1.358 (0.936-1.972)	0.107^a^
AA	31	(14.9)	10	(2.9)	6.625 (3.128-14.034)	0.000^b^
A allele	32.9		19.9			

*Chi-square test;.

a versus GG genotype.

b versus GG genotype.

**Table 5:** Distribution of -448A>G DNMT3A genotypes and associated odds ratio (OR) in relation to age and gender in gastric cancer (GC) cases

Genotype	GC cases (%)	Controls (%)	OR	*P *value
Age(years)				
≤60				
GG	40 (19.2)	80(23.1)	1.0	
AG	40 (19.2)	57 (16.5)	1.404 (0.806-2.444)	0.230
AA	19 (9.1)	4(1.2)	9.500(3.029-29.796)	0.000
>60				
GG	62 (29.8)	138(39.9)	1.0	
AG	35 (16.8)	61 (17.6)	1.277 (0.765-2.132)	0.349
AA	12 (5.8)	6 (1.7)	4.452(1.598-12.404)	0.002
Gender				
Male				
GG	70(33.7)	152(43.9)	1.0	
AG	61(29.3)	82 (23.7)	1.615(1.045-2.498)	0.031
AA	26(12.5)	9 (2.6)	6.273(2.793-14.089)	0.000
Female				
GG	32(15.4)	66(19.1)	1.0	
AG	14(6.7)	36 (10.4)	0.802(0.380-1.694)	0.563
AA	5(2.4)	1 (0.3)	10.313(1.156-91.974)	0.012

Subsequently, in order to explore whether this SNP is also associated with EC, we analysed the frequency of -448A>G. The oesophagus cancer risk related to the DNMT3A -448A>G genotype are shown in Tables 6 and 7. The allele frequency of -448A among EC patients and controls was 21.9% versus 19.9%, respectively. The distributions of -448A>G genotypes in the EC group (GG 63.5%, AG 29.2%, AA 7.3%) were not significantly different from those in the controls. Compared with the reference group, AG+AA genotypes (OR 0.929, 95% CI = 0.569-1.517 *P *= 0.769) and AA homozygotes (OR 2.850, 95% CI = 0.920-8.825 *P *= 0.115) had no greater risk of EC. When the analyses were stratified by the age and gender of patients, only the AA genotype was associated with an increased risk of EC (OR 7.667, 95% CI = 1.300-45.230 *P *= 0.026) at >60 years.

**Table 6:** DNMT3A -448A>G genotype and allele frequencies of case patients and control subjects and their association with oesophagus carcinoma (EC)

Genotype	EC (*n *= 96)		Control subjects (*n *= 241)		Crude odds ratio (95% confidence interval)	*P *value*
				
	No.	(%)	No.	(%)		
- 448A>G						
GG (ref.)	61	(63.5)	149	(61.8)	1.0	
AG	28	(29.2)	86	(35.7)	0.795 (0.473-1.338)	0.388^c^
AA	7	(7.3)	6	(2.5)	2.850 (0.920-8.825)	0.115^d^
A allele	21.9		19.9			

*Chi-squared test;

c versus GG genotype.

d versus GG genotype.

**Table 7:** Distribution of -448A>G DNMT3A genotypes and associated odds ratio (OR) in relation to age and gender in oesophagus carcinoma (EC) cases

Genotype	EC cases (%)	Controls (%)	OR	*P *value
Age(years)				
≤60				
GG	43 (44.8)	80 (33.2)	1.0	
AG	15 (15.6)	57 (23.7)	0.490 (0.248-0.965)	0.037
AA	3 (3.1)	4 (1.7)	1.395 (0.298-6.523)	0.985
>60				
GG	18 (18.7)	69 (28.6)	1.0	
AG	13 (13.5)	29 (12.0)	1.718 (0.746-3.960)	0.201
AA	4 (4.2)	2 (0.8)	7.667(1.300-45.230)	0.026
Gender				
Male				
GG	46 (47.9)	96(39.8)	1.0	
AG	20 (20.8)	59 (24.5)	0.707 (0.382-1.311)	0.271
AA	5 (5.2)	5 (2.1)	2.087 (0.575-7.570)	0.428
Female				
GG	15 (15.6)	53(22.0)	1.0	
AG	8 (8.3)	27 (11.2)	1.047 (0.395-2.776)	0.927
AA	2 (2.1)	1 (0.4)	7.067 (0.599-83.374)	0.140

Thus, our data reveal important evidence that the presence of -448A shows that there is a significance likelihood of carcinogenesis occurring in GC patients but not in EC patients, at least in this Chinese population. The functional polymorphism in the DNMT3A promoter modifies the susceptibility to GC and may be a risk predictor for GC, especially in ≤ 60 year group, which is the main population affected in China.

### Immunohistochemical analysis of DNMT3A in GCs and pericancerous gastric tissues

**Figure 3:**
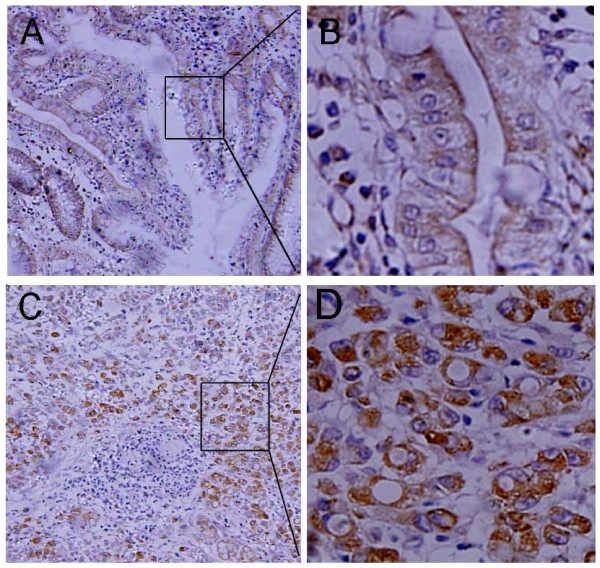
**DNMT3A expression in gastric cancerous and corresponding paracancerous tissues**. DNMT3A is shown as brown and is well distributed in the cytoplasm. (A, B) Immunohistochemical staining of DNMT3A in paracancerous tissues of GCs (A, ×100 and B, ×400). (C, D) Immunohistochemical staining of DNMT3A in cancerous tissues of gastric cancer cases (C, ×100; D ×400).

Immunoreactivity for DNMT3A was detected in the cytoplasm but not in the cell membranes of cancer cells (Figure 3). In order to confirm definitely positive cases from cases with a leaky background level signal, if more than 30% of the cells in a tissue sample exhibited cytoplasm staining, the sample was considered to show positive immunoreactivity. DNMT3A immunoreactivity was detected and overexpressed in 69.64% (39/56) of the patients.

## Discussion

Gastric cancer and esophageal carcinoma are two prevalent tumors and is one of the main deaths in Chinese. In gastric cancers, tumour-suppressor genes (TSGs) are more frequently inactivated by aberrant DNA methylation than by mutations [21]. CpG island hepermethylation of TSGs is associated with a recurrence of early stage oesophageal carcinoma [22-26]. Increased expression of DNMT3A and DNMT3B as *de novo *DNA methyltransferase are common in many tumours which implies the that aberrant DNMT3A and DNMT3B expression are involved in carcinogenesis. Our immunohistochemical data showed that the positive rates of DNMT3A expression in GC tissues were significantly higher than those of para-cancerous tissues (which agrees with a previous study of GC paraffin sections [17]) and therefore shows that SNP of a promoter of a gene may increase the enzyme activity and play a role in the biological process. Our previous results suggested that a polymorphism at position -579 of the DNMT3B promoter was associated with a susceptibility to GC [18] but not EC [27]. To our knowledge, a relationship between the DNMT3A polymorphism and risk of occurrence and progression of GC and EC has not so far been reported.

We hypothesized that polymorphisms of DNMT3A promoter are associated with the risk of cancer. In this case-control study, we report the association between a polymorphism in the human DNMT3A promoter and risk of GC and EC. In 346 healthy control, the allele frequency of -448A is 0.199 and the frequency of -448A>G (AG+AA) is 0.370, implying that it maybe have a potential SNP to evaluate the susceptibility to diseases. A luciferase assay showed that the promoter activity of the construct containing the fragment of DNMT3A of 5'UTR with 448A allele was more than twice as high as the construct containing the 448G allele in the CHO cell line, thus supporting the suggestion that the allele is a functional SNP. The potential mechanism of this association is G >A transition which increases the DNMT3A promoter activity. The relationship between more than twofold overexpression of DNMT3A and an expression of tumour suppressor genes was not confirmed but elevated DNMT3A expression does coincide with some tumour-related genes and includes SFRP5 hypermethylation and transcriptional repression in paired patient biopsies [28,29]. Knockdown DNMT3A expression more than doubles the induced 242 genes in melanoma cells and plays an essential role in melanoma tumourigenesis [30].

In our analysis of the DNMT3A gene promoter, -448A>G polymorphism among the GCs, ECs and the control group drawn from a Chinese population, it was shown that -448A allele and AA genotype are distributed in the all three study groups. We found that the A variant genotype was associated with a significantly increased risk of GC. AA genotype was associated with a significantly increased risk of GC at ≤ 60 years and if the participants were female. Further studies are needed in order to elucidate the role of DNMT3A variants in the expression level of DNMT3A in GC patients and the function of the DNA methylaton. However, this polymorphism is not associated with a susceptibility to EC. Although the analyses were stratified by age and gender of patients, only the AA genotype is associated with an increased risk of EC at > 60 years.

The present study provides evidence that a SNP in the DNMT3A promoter region may modify the risk of GC but not of oesophagus carcinoma. Genetic studies are required in order to explain why individuals exposed to similar risk factors develop different kinds of tumours. Different genetics factors, including tumour suppressor genes and silencing catalyzed by *de novo *methylatransferases, were involved in the tumourigenesis of different types of tumours. Although it has been reported that several tumour suppressor genes, such as p16 [31,32], E-cadherin [9,33] TIMP3 [34], DLC1 [35,36] and RUNX3 [37,38] silence GC and EC, only MINT25, RORA, GDNF, ADAM23, PRDM5, MLF1 showed frequent differential methylation in GC [39] which implies that different genetic and epigenetic mechanism are involved in the development and progression of these two tumour types. Then, DNMT3A expression was detected in AA, GA, and GG variants genotype carriers in GC and EC patients (Additional File 1). There was higher DNMT3A expression in GA genotype carriers of GCs but lower DNMT3A in GA carriers of ECs. AA homozygote was not found in the detected cases, possibly because the examined group was not large enough. These data suggested that *DNMT3A *may play a role in the progression of gastric cancer and this finding needs to be confirmed by a larger study.

In addition, as genetic polymorphisms often vary in tissues in different ethnic groups, further studies are needed in order to clarify the association of the DNMT3A polymorphism with GC in diverse ethnic populations. Future studies of other DNMT3A sequence variants and their biologic function are also needed in order to understand the role of DNMT3A polymorphisms in determining the risk of cancer. The current study suggested that at least some DNMT3A polymorphisms, including the DNMT3A promoter SNP, may play different roles in the development and progression of these two tumor types. The importance of the DNMT3A gene in the regulation of DNA methylation and gene expression makes it attractive for further study of other cancers and it could be a useful marker for epidemiological study.

## Conclusion

In conclusion, our study provides the first evidence that this newly identified polymorphism of the DNMT3A promoter is significantly associated with an increased risk of GC in this study population. The potential mechanism for the higher risk associated with the variants may be an increased activity of G > A. These results suggest that the polymorphism of DNMT3A could be used as an important marker of genetic susceptibility to GC, although additional studies using larger sample sizes are required to confirm our findings.

## Abbreviations

CHO: Chinese hamster ovary; DNMT: DNA-methyltransferase; EB: ethidium bromide; EC: oesophagus carcinoma; EDTA: ethylenediaminetetra-acetic acid; GC: gastric cancer; PCR: polymerase chain reaction; RFLP: restriction fragment length polymorphism; SNP: single nucleotide polymorphism; TSG: tumour-suppressor gene.

## Competing interests

The authors declare that they have no competing interests.

## Authors' contributions

HF designed and carried out part of the DNMT3A -448A>G polymorphism analysis and wrote the manuscript; DSL carried out DNMT3A -448A>G polymorphism analysis in GC patients and controls. QXW and ZJZ performed the immunohistochemistry analysis of DNMT3A in GC patients. XMQ and FZ collected patient's clinical data and performed mRNA expression of DNMT3A in GC and EC patients (in Additional File 2). FCQ, XWS and YWS performed DNMT3A -448A>G polymorphism analysis in EC research and analysed the data. WX contributed to the design and the preparation of the paper. HF and DSL contributed equally to this work. All authors read and approved the final manuscript.

## Pre-publication history

The pre-publication history for this paper can be accessed here:

http://www.biomedcentral.com/1741-7015/8/12/prepub

## Supplementary Material

Additional file 1 Figure S1 Click here for file

Additional file 2 Supplementary information. Click here for file
