# Supplementary material for: A functional polymorphism in the DNA methyltransferase-3A promoter modifies the susceptibility in gastric cancer but not in esophageal carcinoma
Source: BMC Med. 2010 Feb 3;8:12. doi: 10.1186/1741-7015-8-12 (PMC2829483; doi:10.1186/1741-7015-8-12)
Supplement: Additional file 1 — Figure S1 [file 1741-7015-8-12-S1.DOC]

**Supplementary**

**Material and Methods**

***Study Subjects***

Tumor tissues and adjacent non-cancerous tissue from 29 GCs and 20 ECs were obtained from the First Hospital of Nanjing, affiliated with Nanjing Medical University, from May 2008 to March 2009. All of the samples were diagnosed by a single pathologist, and appropriate consent was obtained from each patient.

***Quantitative RT-PCR detcted the DNMA3A expression***

Complementary DNA was subject to quantitative real-time polymerase chain reaction (qPCR) for *DNMT3A* expression using a SYBR Green PCR Kit (TaKaRa, Japan) under the following cycling conditions: PCR reactions were performed in a 50 μL volume with 5U polymerase (TaKaRa，Japan ) and cDNA samples equivalent to 1 ng of RNA. SYBR Green at a dilution of 1:20,000 was included in each reaction for the relative quantification using the ABI 7300 sequence detection system (Applied Biosystems，USA). To normalize the cDNA input among samples, *β -actin* was quantified and used as an endogenous standard. The relative level of expression of DNMT3A among the different tissues was then calculated in relation to the amount of *β –actin* *(*ABI PRISM 7300 Detection System, USA). Quantitative PCRs were performed in duplicate for each sample-primer set, and the mean of the two experiments was used as the relative quantification value.

**Results**

The mRNA expression level of the *DNMT3A* was detected by qPCR in tumor and non-tumor tissues specimens from 29 GC patients and 20 EC patients, respectively. There was higher DNMT3A expression in GA genotype carriers of GCs, however, lower DNMT3A in GA carriers of ECs. AA homozygote wasn’t found in detected cases. These data suggested that *DNMT3A* may play a role in the progression of gastric cancer, and this finding needs to be confirmed by a larger study.

Figure 1

Relative *DNMT3A* mRNA expression levels were determined by real-time quantitative PCR. The top and bottom horizontal lines of the box indicate the 25th and 75th percentiles, respectively. The bold lines within the box indicate the median values. The top and bottom horizontal bars indicate the maximum and minimum values respectively. (A) The level of *DNMT3A* in gastric tumor tissues of GA carriers was higher than that of GG carriers. (B) There was no *DNMT3A* higher expression pattern in esophagus tumor tissues of GA carriers compared with GG carriers.
