# Supplementary figures and images for: A functional polymorphism in the DNA methyltransferase-3A promoter modifies the susceptibility in gastric cancer but not in esophageal carcinoma
Source: BMC Med. 2010 Feb 3;8:12. doi: 10.1186/1741-7015-8-12 (PMC2829483; doi:10.1186/1741-7015-8-12)

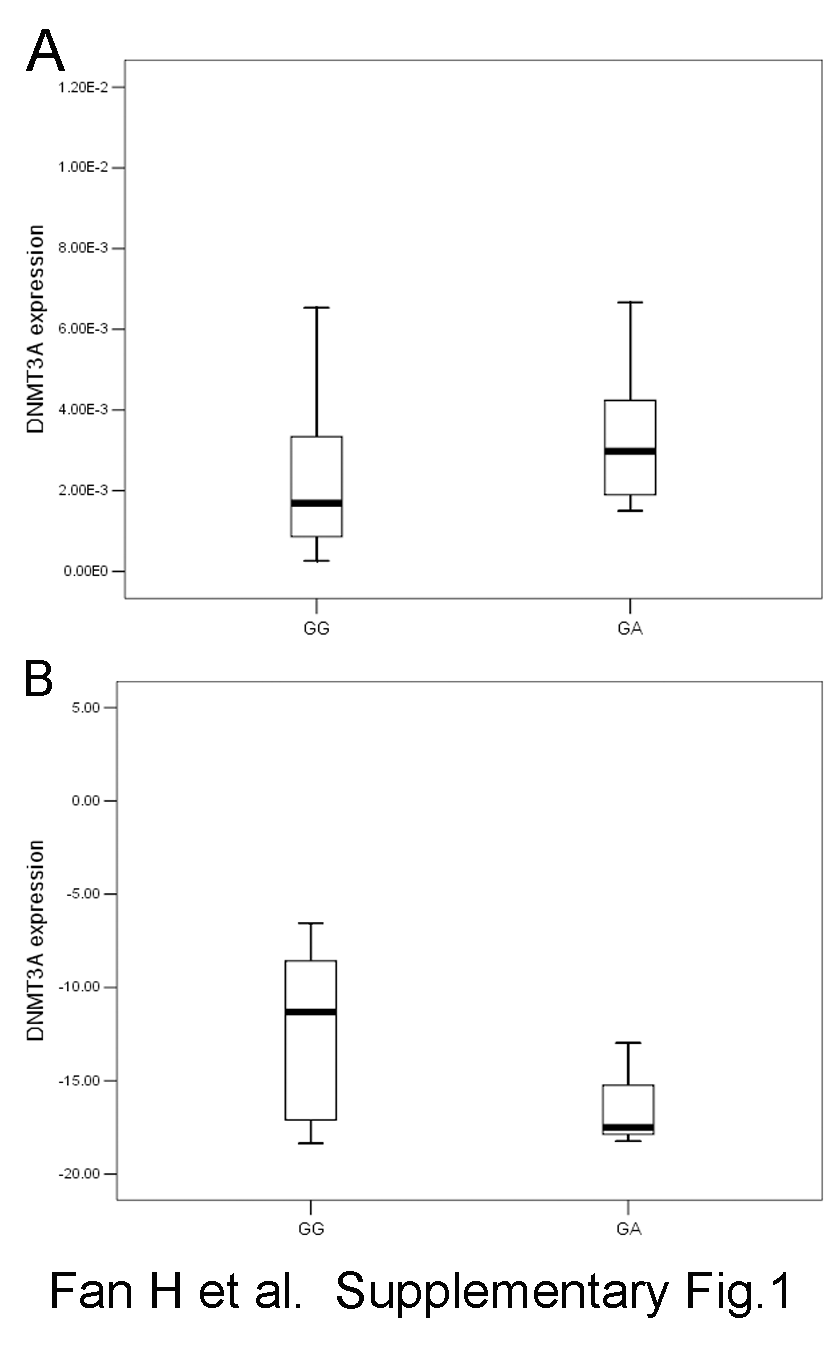

Supplement: Additional file 2 — Supplementary information. [file 1741-7015-8-12-S2.TIFF]
